# The effect of goal-directed therapy on mortality in patients with sepsis - earlier is better: a meta-analysis of randomized controlled trials

**DOI:** 10.1186/s13054-014-0570-5

**Published:** 2014-10-20

**Authors:** Wan-Jie Gu, Fei Wang, Jan Bakker, Lu Tang, Jing-Chen Liu

**Affiliations:** Department of Anaesthesiology, the First Affiliated Hospital, Guangxi Medical University, 22 Shuangyong Road, Nanning, 530021 China; Department of Anaesthesiology, General Hospital of Jinan Military Command, 25 Shifan Road, Jinan, 250031 China; Department of Intensive Care Adults, Erasmus MC University Medical Centre, PO Box 2040, 3000 Rotterdam, CA the Netherlands

## Abstract

**Introduction:**

The Surviving Sepsis Campaign guidelines recommend goal-directed therapy (GDT) for the early resuscitation of patients with sepsis. However, the findings of the ProCESS (Protocolized Care for Early Septic Shock) trial showed no benefit from GDT for reducing mortality rates in early septic shock. We performed a meta-analysis to integrate these findings with existing literature on this topic and evaluate the effect of GDT on mortality due to sepsis.

**Methods:**

We searched the PubMed, Embase and CENTRAL (Cochrane Central Register of Controlled Trials) databases and reference lists of extracted articles. Randomized controlled trials comparing GDT with standard therapy or usual care in patients with sepsis were included. The prespecified primary outcome was overall mortality.

**Results:**

In total, 13 trials involving 2,525 adult patients were included. GDT significantly reduced overall mortality in the random-effects model (relative risk (RR), 0.83; 95% confidence interval (CI), 0.71 to 0.96; *P* =0.01; *I*^2^ = 56%). Predefined subgroup analysis according to the timing of GDT for resuscitation suggested that a mortality benefit was seen only in the subgroup of early GDT within the first 6 hours (seven trials; RR, 0.77; 95% CI, 0.67 to 0.89; *P* =0.0004; *I*^2^ = 40%), but not in the subgroup with late or unclear timing of GDT (six trials; RR, 0.92; 95% CI, 0.69 to 1.24; *P* =0.59; *I*^2^ = 56%). GDT was significantly associated with the use of dobutamine (five trials; RR, 2.71; 95% CI, 1.20 to 6.10; *P* =0.02).

**Conclusions:**

The results of the present meta-analysis suggest that GDT significantly reduces overall mortality in patients with sepsis, especially when initiated early. However, owing to the variable quality of the studies, strong and definitive recommendations cannot be made.

**Electronic supplementary material:**

The online version of this article (doi:10.1186/s13054-014-0570-5) contains supplementary material, which is available to authorized users.

## Introduction

Sepsis is a systemic response to infection, which may progress to severe sepsis and septic shock [1]. Severe sepsis and septic shock represent global problems with a high economic burden. In the United States, more than 750,000 people experience severe sepsis each year, with a short-term mortality of 20% to 30%, reaching up to 50% when shock is present [2,3]. Therefore, numerous therapeutic strategies that aimed at reducing mortality in these patients have been investigated. However, most of them have not led to significant reductions in mortality [4]. Goal-directed therapy (GDT) has been shown to substantially improve clinical outcomes in surgical patients [5]. Two important aspects of a GDT protocol include early initiation of the therapeutic measures, together with specific (hemodynamic) targets. The Surviving Sepsis Campaign guidelines recommend GDT for the early resuscitation of patients with sepsis [1], which is based largely upon the results of the Rivers *et al*. trial, in which the researchers reported a 16% absolute reduction in mortality among patients with severe sepsis or septic shock who received early GDT compared to standard therapy [6]. However, in a recent study, this approach was challenged [7], with no benefit shown when GDT was compared to standard therapy. However, initiating therapy early rather than late in the course of critical illness remains a logical clinical goal. In the context of this situation, we systematically reviewed all trials of GDT in patients with sepsis and performed a meta-analysis, focusing on the early initiation of the protocol and its effect on mortality.

## Methods

Ethical approval and patient consent were not required, because we conducted a meta-analysis of previously published studies. We followed the recommendations of the *Cochrane Handbook for Systematic Reviews of Interventions* to carry out the study [8] and follow the PRISMA (Preferred Reporting Items for Systematic Reviews and Meta-Analyses) statement to report our meta-analysis [9].

### Search strategy

Electronic searches were conducted in the PubMed, Embase and Cochrane Central Register of Controlled Trials (CENTRAL) databases. Search terms included “goal directed,” “goal oriented,” “goal target,” “cardiac output,” “cardiac index,” “oxygen delivery,” “oxygen consumption,” “cardiac volume,” “stroke volume,” “fluid therapy,” “fluid loading,” “fluid administration,” “optimization,” “optimization,” “supranormal” and “sepsis,” “severe sepsis,” “septic shock,” “septicemia,” “septicaemia,” “pyohemia,” “pyaemia” and “pyemia.” There was no language restriction placed on the searches. Each database was searched from inception to April 2014. Additionally, reference lists in the articles chosen for inclusion, and the reference lists of previous reviews were screened to identify other potentially eligible trials.

### Inclusion criteria

We included trials with the following characteristics:

1. *Population*: Adult patients with one or more of the following characteristics were eligible for inclusion: sepsis, severe sepsis or septic shock. *Adults* were defined as being of a legal age for consent in the country where the trial was conducted. Studies that included sepsis secondary to noninfectious causes were excluded.
2. *Intervention*: The intervention had to be *GDT*, defined as an explicit protocol encompassing the use of hemodynamic monitoring and manipulation of hemodynamic parameters to achieve predetermined hemodynamic endpoints.
3. *Control*: The control group had to have received standard therapy or usual care.
4. *Outcomes*: The overall mortality rate had to be the outcome measured.
5. *Type of study*: The studies had to be randomized controlled trials (RCTs).

We included studies that randomized a mixed population of critically ill patients when a septic subpopulation that met our inclusion criteria was defined; that is, the patients with sepsis constituted a subgroup of the trial population.

### Data extraction

We extracted data using a standardized data collection form. Discrepancies in collected data were addressed through team consensus. The following information was extracted from each trial: first author, year of publication, number of patients (GDT and control), study population, clinical setting, goals in GDT and control groups, timing of GDT, mortality endpoint, study design (patient selection and concealment) and outcome data (overall mortality and dobutamine use).

### Outcomes and definitions

The prespecified primary outcome was overall mortality. If the study authors reported mortality at one time point, we used the only data used for analysis. If the study authors reported mortality at more than one time point, we used hospital mortality preferentially. Secondary outcomes included overall mortality in the early-initiated GDT (that is, within the first 6 hours) versus GDT and dobutamine use initiated later.

### Assessment of risk of bias

We used the Cochrane Collaboration tool to assess the risk of bias of individual study and with bias domains across studies [8,10]. Two investigators (WJG and FW) subjectively reviewed all studies and assigned a value of “high,” “low” or “unclear” to the following domains: random sequence generation, allocation concealment, blinding of participants and personnel, blinding of outcome assessment, incomplete outcome data, selective reporting and other bias. Trials with high risk of bias for any one or more key domains were considered to be at high risk of bias. Trials with low risk of bias for all key domains were considered to be at low risk of bias. Otherwise, they were considered to have an unclear risk of bias [10].

### Statistical analysis

We estimated the relative risk (RR) with 95% confidence interval (CI) for dichotomous outcomes. Statistical heterogeneity across studies was tested by using the *I*^2^ statistic [11,12]. Heterogeneity was suggested if the *P*-value was ≤0.10. *I*^2^ values of 0 to 24.9%, 25% to 49.9%, 50% to 74.9% and 75% to 100% were considered zero, low, moderate and high thresholds for statistical heterogeneity, respectively [11,12]. Clinical heterogeneity could not be excluded, so the more conservative random-effects model [13] (Mantel–Haenszel method) was used. Predefined subgroup analysis was conducted according to the timing of GDT for resuscitation (early being within the first 6 hours versus late or unclear being outside the first 6 hours or unclear timing). In addition, we performed *post hoc* subgroup analyses according to risk of bias (low versus unclear), sample size (≥100 versus <100) and setting (emergency department versus intensive care unit). We further investigated the influence of a single study on the overall pooled estimate by omitting one study in each step. The potential for bias was assessed by inspection of a funnel plot and Egger’s test [14]. The results were considered statistically significant at two-sided *P*-values <0.05. All statistical analyses were performed using RevMan 5.2 software (The Nordic Cochrane Centre, Copenhagen, Denmark).

### Quality of evidence

We evaluated the quality of the evidence by using the Grades of Recommendation, Assessment, Development and Evaluation (GRADE) approach [15]. In addition, the GRADEprofiler 3.6 software (The Nordic Cochrane Centre) was used to create the evidence profile. The GRADE Working Group grades of evidence used were as follows:

1. *High quality*: Further research is very unlikely to change our confidence in the estimate of effect.
2. *Moderate quality*: Further research is likely to have an important impact on our confidence in the estimate of effect and may change the estimate.
3. *Low quality*: Further research is very likely to have an important impact on our confidence in the estimate of effect and is likely to change the estimate.
4. *Very low quality*: We are very uncertain about the estimate.

## Results

**Figure 1:**
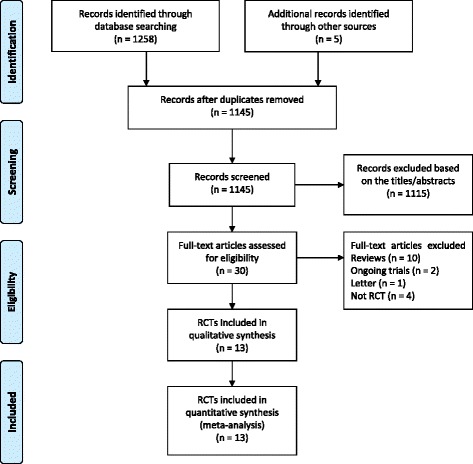
**Flow diagram showing results of search and reasons for exclusion of studies.** RCT, Randomized controlled trial.

In the initial search, we identified 1,263 records. After examination of the titles and abstracts, there were 30 potentially eligible studies assessed for inclusion. After application of the inclusion criteria, 13 RCTs [6,7,16-26] were included in the meta-analysis. The study flow diagram, including the reasons for exclusion of studies, is shown in Figure 1.

### Study characteristics

The characteristics of the included trials are presented in Table 1. These trials were published between 1992 and 2014. The sample size of the trials ranged from 34 to 895, with a total of 2,525 patients comprising 1,299 in the GDT group and 1,226 in the control group. Eleven trials were conducted in the intensive care unit [16-26], and the remaining two were conducted in the emergency department [6,7]. Four trials were published in Chinese [23-26], and the other nine trials were in English [6,7,16-22]. Early GDT for resuscitation within the first 6 hours was reported in seven trials [6,7,16,21,22,24-26], late GDT for resuscitation outside the first 6 hours was assessed in one trial [23] and unclear timing of GDT was described in five trials [17-21]. Overall mortality was reported in all trials [6,7,16-26], and dobutamine use was described in eight of them [6,7,16-19,21,22].

**Table 1:** **Characteristics of included randomized controlled trials** ^**a**^

**Study**	**Year**	**No. of patients with sepsis (GDT/control)**	**Study population**	**Clinical setting**	**Goals in GDT group**	**Goals in control group**	**Timing of GDT**	**Mortality endpoint**
Tuchschmidt *et al*. [16]	1992	51 (26/25)	Adult patients with septic shock	ICU	CI ≥6 L/min/m^2^	CI ≥3 L/min/m^2^	Within the first 6 hr	14 days
					SBP ≥90 mmHg	SBP ≥90 mmHg		
Yu *et al*. [17]	1993	52 (30/22)	Adult patients with sepsis or septic shock	ICU	DO_2_I >600 ml/min/m^2^	DO_2_I 450 to 550 ml/min/m^2^	Unclear	30 days
			(septic subpopulation)		SBP >100 mmHg	SBP >100 mmHg		
Hayes *et al*. [18]	1994	47 (24/23)	Adult patients with septic shock	ICU	CI ≥4.5 L/min/m^2^	Usual care	Unclear	Hospital
			(septic subpopulation)		DO_2_ 600 ml/min/m^2^			
					VO_2_ > 170 ml/min/m^2^			
Gattinoni *et al*. [19]	1995	181 (124/57)	Adult patients with septic shock or septic syndrome	ICU	CI ≥4.5 L/min/m^2^ or SvO_2_ ≥ 70%	CI 2.5 to 3.5 L/min/m^2^	Unclear	ICU
						MAP ≥65 mmHg		
			(septic subpopulation)		MAP ≥65 mmHg	CVP 8 to 12 mmHg		
					CVP 8 to 12 mmHg	UO ≥0.5 ml/kg/hr		
					UO ≥0.5 ml/kg/h			
Yu *et al*. [20]	1998	87 (58/29)	Adult patients with sepsis, severe sepsis or septic shock	ICU	DO_2_I >600 ml/min/m^2^	DO_2_I 450 to 550 ml/min/m^2^	Unclear	ICU
					SBP ≥100 mmHg	SBP ≥100 mmHg		
			(septic subpopulation)		SvO_2_ > 65%	SvO_2_ > 65%		
					UO >50 ml/hr	UO >50 ml/hr		
Alía *et al*. [21]	1999	63 (31/32)	Adult patients with severe sepsis or septic shock	ICU	DO_2_I >600 ml/min/m^2^	DO_2_I >330 ml/min/m^2^	Unclear	ICU
					MAP >60 mmHg	MAP >60 mmHg		
Rivers *et al*. [6]	2001	263 (130/133)	Adult patients with severe sepsis, septic shock or sepsis syndrome	ED	SvO_2_ ≥ 70%	CVP 8 to 12 mmHg	Within the first 6 hr	Hospital
					CVP 8 to 12 mmHg	MAP 65 to 90 mmHg		
					MAP 65 to 90 mmHg	UO ≥0.5 ml/kg/hr		
					UO ≥0.5 ml/kg/hr			
Lin *et al*. [22]	2006	224 (108/116)	Adult patients with septic shock	ICU	CVP 8 to 12 mmHg	Usual care	Within the first 6 hr	Hospital
					MAP ≥65 mmHg			
					UO ≥0.5 ml/kg/hr			
Wang *et al*. [23]	2006	34 (16/17)	Adult patients with septic shock	ICU	SvO_2_ ≥ 70%	MAP ≥65 mmHg	Within the first 6 to 10 hr	14 days
					CVP 8 to 12 mmHg	UO ≥0.5 ml/kg/hr		
					MAP ≥65 mmHg			
					UO ≥0.5 ml/kg/hr			
Chen *et al*. [24]	2007	123 (58/65)	Adult patients with severe sepsis	ICU	ScvO_2_ ≥ 70%	CVP 8 to 12 mmHg	Within the first 6 hr	ICU
			(septic subpopulation)		CVP 8 to 12 mmHg	MAP ≥65 mmHg		
					MAP ≥65 mmHg	UO ≥0.5 ml/kg/hr		
					UO ≥0.5 ml/kg/hr			
He *et al*. [25]	2007	203 (98/105)	Adult patients with septic shock	ICU	ScvO_2_ or SvO_2_ ≥ 70%	Usual care	Within the first 6 hr	Hospital
					CVP 8 to 12 mmHg			
					MAP ≥65 mmHg			
					UO ≥0.5 ml/kg/hr			
Yan *et al*. [26]	2010	303 (157/146)	Adult patients with severe sepsis or septic shock	ICU	ScvO_2_ ≥ 70%	CVP 8 to 12 mmHg	Within the first 6 hr	ICU
					CVP 8 to 12 mmHg	SBP >90 mmHg		
					SBP >90 mmHg	MAP ≥65 mmHg		
					MAP ≥65 mmHg	UO ≥0.5 ml/kg/hr		
					UO ≥0.5 ml/kg/hr			
ProCESS [7]	2014	895 (439/456)	Adult patients with septic shock	ED	ScvO_2_ ≥ 70%	Usual care	Within the first 6 hr	Hospital
					CVP 8 to 12 mmHg			
					MAP 65 to 90 mmHg			
					UO ≥0.5 ml/kg/hr			

^a^CI, Cardiac index; CVP, Central venous pressure; DO_2_, Oxygen delivery; DO_2_I, Oxygen delivery index; ED, Emergency department; GDT, Goal-directed therapy; ICU, Intensive care unit; MAP, Mean arterial pressure; ProCESS, Protocolized Care for Early Septic Shock; SIRS, Systemic inflammatory response syndrome; SBP, Systolic blood pressure; ScvO_2_, Central venous oxygen saturation; SvO_2_, Mixed venous oxygen saturation; UO, Urine output; VO_2_, Oxygen consumption.

### Risk of bias in included studies

**Figure 2:**
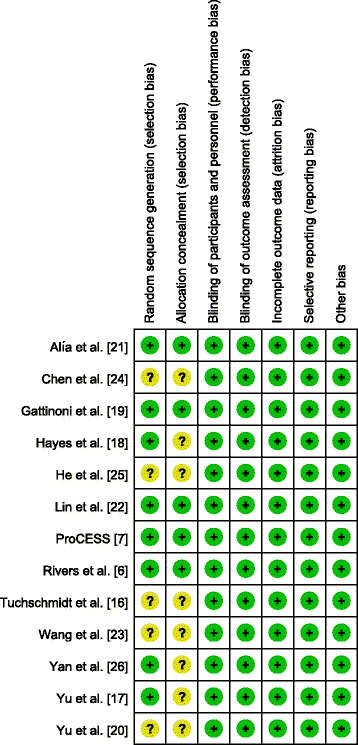
**Risk of bias summary.**

The details of risk of bias are summarized in Figure 2. Five trials were judged to be at low risk of bias [6,7,19,21,22], and eight trials were judged to be at unclear risk of bias [16-18,20,23-26]. Adequate randomized sequences were generated in eight trials [6,7,17-19,21,22,26], and the investigators in five trials reported appropriate allocation concealment [6,7,19,21,22]. Among the 13 RCTs, none were double-blinded. However, blinding of patients and clinicians was extremely difficult in these trials to evaluate a complex intervention such as a GDT protocol, and we judged that the primary outcome (that is, overall mortality) was not likely to be influenced by lack of blinding.

### Primary outcome: overall mortality

**Figure 3:**
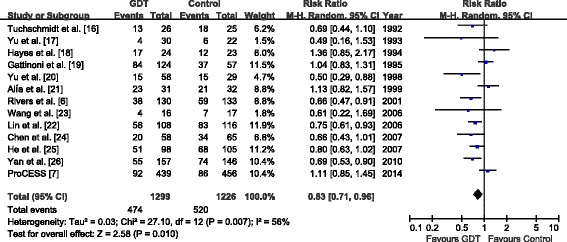
**Forest plot of the effect goal-directed therapy on overall mortality.** GDT, Goal-directed therapy.

Mortality data were available in all 13 included trials [6,7,16-26]. The overall mortality data in the GDT and control groups were 474 (36.5%) of 1,299 and 520 (42.4%) of 1,226, respectively. Overall, GDT significantly reduced overall mortality in the random-effects model (RR, 0.83; 95% CI, 0.71 to 0.96; *P* =0.01; *I*^2^ = 56%) (Figure 3). Further exclusion of any single study did not alter the overall combined RR, which ranged from 0.80 (95% CI, 0.69 to 0.93) to 0.85 (95% CI, 0.73 to 0.98). The results of subgroup analyses are presented in Table 2.

**Table 2:** **Subgroup analyses of overall mortality** ^**a**^

**Subgroups**	**No. of studies**	**No. of patients**	**RR (95% CI)**	***P***-**value**	***I*** ^**2**^ **(%)**
All trials [6,7,16-26]	13	2,525	0.83 (0.71 to 0.96)	0.01	56
GDT timing					
Early [6,7,16,22,24-26]	7	2,062	0.77 (0.67 to 0.89)	0.0004	40
Late or unclear [17-21,23]	6	463	0.92 (0.69 to 1.24)	0.59	56
Risk of bias					
Low [6,7,19,21,22]	5	1,626	0.92 (0.75 to 1.13)	0.42	67
Unclear [16-18,20,23-26]	8	899	0.74 (0.62 to 0.89)	0.002	31
Sample size					
≥100 [6,7,19,22,24-26]	7	2,192	0.82 (0.70 to 0.95)	0.01	59
<100 [16-18,20,21,23]	6	333	0.81 (0.56 to 1.17)	0.27	61
Setting					
ED [6,7]	2	1,158	0.86 (0.52 to 1.44)	0.52	83
ICU [16-26]	11	1,367	0.81 (0.69 to 0.96)	0.01	53

^a^CI, Confidence interval; ED, Emergency department; GDT, Goal-directed therapy; ICU, Intensive care medicine; RR, Relative risk.

### Secondary outcomes

**Figure 4:**
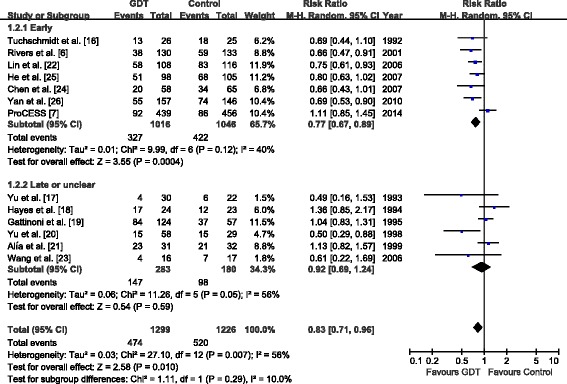
**Forest plot of the effect goal-directed therapy on overall mortality according to the timing of treatment.** GDT, Goal-directed therapy.

Predefined subgroup analysis according to the timing of GDT for resuscitation suggested that a mortality benefit was seen only in the subgroup in early GDT within the first 6 hours (seven trials; RR, 0.77; 95% CI, 0.67 to 0.89; *P* =0.0004; *I*^2^ = 40%) (Figure 4), but not in the subgroup with late or unclear timing of GDT (six trials; RR, 0.92; 95% CI, 0.69 to 1.24; *P* =0.59; *I*^2^ = 56%) (Figure 4).

In five trials, the investigators reported available data on dobutamine use [6,7,16,21,22]. In those trials, GDT was significantly associated with dobutamine use (RR, 2.71; 95% CI, 1.20 to 6.10; *P* =0.02; *I*^2^ = 86%).

### Publication bias

**Figure 5:**
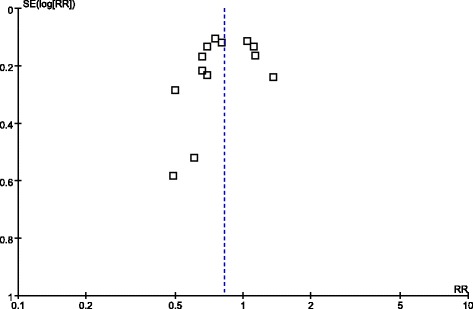
**Funnel plot of the effect goal-directed therapy on overall mortality.** GDT, Goal-directed therapy.

We detected no evidence of publication bias by assessing funnel plot either visually (Figure 5) or statistically (*P* =0.367 by Egger test).

### GRADE profile evidence

We found that GRADE Working Group grades of evidence were low for overall mortality, moderate for mortality in early GDT (within the first 6 hours for resuscitation) and very low for dobutamine use. An additional .doc file shows these data in more detail (see Additional file 1).

## Discussion

Our meta-analysis of 13 RCTs showed that GDT was associated with a 17% RR reduction on overall mortality in patients with sepsis. This mortality benefit was present in studies in which GDT was started early, but not when initiated late or when the timing of GDT was unclear. In addition, GDT was significantly associated with dobutamine use.

Despite these findings, the effect of GDT remains a matter of debate, as the most recent and largest trial included in this meta-analysis [7] did not show a difference in mortality, in contrast to many of the preceding studies. This could be due to the effect of the rapid acceptance of the principal interventions of the Rivers *et al*. study [6] and the subsequent Surviving Sepsis Campaign guidelines, which encompassed all elements of the Rivers study protocol. This is illustrated by the fact that, in the ProCESS (Protocolized Care for Early Septic Shock) trial, all groups received, on average, more than 2 L of fluid prior to randomization and more than 75% of patients received antibiotics before randomization. In addition, the mortality rate was much lower in the ProCESS trial than in preceding trials, possibly reflecting the effect of early diagnosis, fluid resuscitation and initiation of antibiotics on mortality. As the ProCESS trial, like the Rivers *et al*. study, enrolled patients with sepsis in the emergency department, this effect may have been prominent [27].

Although the current evidence supports the early use of GDT to improve outcomes in patients with sepsis, the optimal goals remain uncertain. Currently, the Surviving Sepsis Campaign guidelines recommend the use of central venous pressure (CVP), mean arterial pressure, urine output and central venous oxygen saturation (ScvO_2_) as resuscitation goals. However, many of these recommendations have been questioned in recent studies. A recent study [28] was designed to compare the use of lactate clearance to ScvO_2_ as a goal of early (up to 6 hours) sepsis resuscitation. No significant difference in mortality was found (17% in the lactate clearance group versus 23% in ScvO_2_ group). However, when both normalization of ScvO_2_ and a rapid decrease in lactate levels were applied as therapeutic goals in the early resuscitation of a mixed group of critically ill patients, including a large subgroup of sepsis patients, mortality was significantly reduced [29]. In addition, in a recent retrospective study, researchers questioned the CVP endpoint in sepsis resuscitation [30].

Further research is needed before strong and definitive recommendations can be made regarding the effect of GDT for resuscitation of patients with sepsis. There are currently at least two ongoing RCTs of GDT in patients with sepsis: the Australasian Resuscitation in Sepsis Evaluation (ARISE) trial in Australia (ClinicalTrials.gov ID: NCT00975793) and the Protocolised Management in Sepsis (ProMISe) trial in the United Kingdom (Current Controlled Trials number: ISRCTN36307479) [31]. The results of these ongoing trials should provide further guidance as to the effect of GDT for resuscitation of patients with sepsis.

A major strength of the present meta-analysis is its compliance with the Cochrane handbook methodology recommendations. We conducted an exhaustive literature search that included non-English-language articles. Two authors independently screened all references, included eligible trials, extracted data information, assessed risk of bias and performed statistical analyses. Moreover, we followed the PRISMA statement to report this meta-analysis and evaluated the quality of the evidence by using the GRADE approach.

Because early fluid resuscitation is vital in patients with sepsis, we performed predefined subgroup analyses according to the timing of GDT. We also performed *post hoc* subgroup analyses according to risk of bias, disease severity, sample size and publication date. These subgroup analyses based on assessment of bias and clinically relevant groups may help health care professionals in clinical decision-making.

Our analysis also has several limitations that must be taken into consideration when interpreting the results. First, most of the included trials had a high risk of bias (Figure 2). The potential importance of this issue is highlighted by the fact that predefined subgroup analysis comparing mortality estimates between trials with low versus unclear risk of bias suggested the mortality benefit is not clearly apparent among the trials with low risk of bias, although this subgroup difference was not statistically significant (*P* =0.90). Second, there were some differences in the target populations and protocols of GDT of each study. These factors may have a potential impact on our results and may preclude firm conclusions. Third, different endpoints were used for mortality evaluation. Because this study was a study-level meta-analysis, individual patient data were not included in the analysis; thus, we could not adjust for patient-level confounders.

## Conclusions

The evidence suggests that GDT significantly reduces overall mortality in patients with sepsis, especially when initiated early (within the first 6 hours of admission). Until the results of ongoing randomized controlled trials are known, strong and definitive recommendations cannot be made regarding the effect of GDT for resuscitation of patients with sepsis.

## Key messages

- The Surviving Sepsis Campaign guidelines recommend GDT for the early resuscitation of patients with sepsis, but controversies about its effect remain.
- The recent ProCESS trial has shown no mortality benefit from GDT in early septic shock.
- The current evidence, in the aggregate, suggests that GDT significantly reduces overall mortality in patients with sepsis, especially when initiated early (within the first 6 hours of admission).
- Further research is needed before strong and definitive recommendations can be made regarding the effect of GDT for resuscitation of patients with sepsis, and the optimal goals remain uncertain.

## Additional file

Additional file 1:
**GRADE summary of findings.**

## Abbreviations

ARISE: Australasian Resuscitation in Sepsis Evaluation
CI: Confidence interval
CVP: Central venous pressure
GDT: Goal-directed therapy
GRADE: Grades of Recommendation, Assessment, Development and Evaluation
MAP: Mean arterial pressure
PRISMA: Preferred reporting items for systematic reviews and meta-analyses
ProMISe: Protocolised management in sepsis
RCT: Randomized controlled trial
RR: Relative risk
ScvO_2_: Central venous oxygen saturation
