# Supplementary material for: The effect of goal-directed therapy on mortality in patients with sepsis - earlier is better: a meta-analysis of randomized controlled trials
Source: Crit Care. 2014 Oct 20;18(5):570. doi: 10.1186/s13054-014-0570-5 (PMC4232636; doi:10.1186/s13054-014-0570-5)
Supplement: Additional file 1: — GRADE summary of findings. [file 13054_2014_570_MOESM1_ESM.doc]

**Additional file 1 GRADE summary of findings**

| **Goal-directed therapy compared to control for sepsis** | | | | | |
| --- | --- | --- | --- | --- | --- |
| **Patient or population:** Adult patients with sepsis | | | | | |
| **Intervention:** Goal-directed therapy | | | | | |
| **Comparison:** Control | | | | | |
| **Outcomes** | **Illustrative comparative risks* (95% CI)** | | **Relative effect** | **No of Participants** | **Quality of the evidence** |
|  | Assumed risk | Corresponding risk | **(95% CI)** | **(studies)** | **(GRADE)** |
|  | **Control** | **GD** |  |  |  |
| **Mortality** | **Study population** | | **RR 0.83** | 2525 | ⊕⊕⊝⊝ |
|  | **424 per 1000** | **352 per 1000**  (301 to 407) |  |  |  |
|  | **Moderate** | | (0.71 to 0.96) | (13 studies) | **low**^1,2^ |
|  | **522 per 1000** | **433 per 1000**  (371 to 501) |  |  |  |
| **Mortality in EGDT (within the first 6 hours for resuscitation)** | **Study population** | | **RR 0.77** | 2062 | ⊕⊕⊕⊝ |
|  | **403 per 1000** | **311 per 1000**  (270 to 359) |  |  |  |
|  | **Moderate** | | (0.67 to 0.89) | (7 studies) | **moderate**^3^ |
|  | **523 per 1000** | **403 per 1000**  (350 to 465) |  |  |  |
| **Dobutamine use** | **Study population** | | **RR 2.71** | 1942 | ⊕⊝⊝⊝ |
|  | **41 per 1000** | **110 per 1000**  (49 to 247) |  |  |  |
|  | **Moderate** | | (1.2 to 6.1) | (5 studies) | **very low**^4,5^ |
|  | **138 per 1000** | **374 per 1000**  (166 to 842) |  |  |  |

*The basis for the **assumed risk** (e.g. the median control group risk across studies) is provided in footnotes. The **corresponding risk** (and its 95% confidence interval) is based on the assumed risk in the comparison group and the **relative effect** of the intervention (and its 95% CI). **CI:** Confidence interval; **EGDT:** early goal-directed therapy; **GDT:** goal-directed therapy; **RR:** Risk ratio.

GRADE Working Group grades of evidence.

**High quality:** Further research is very unlikely to change our confidence in the estimate of effect.

**Moderate quality:** Further research is likely to have an important impact on our confidence in the estimate of effect and may change the estimate.

**Low quality:** Further research is very likely to have an important impact on our confidence in the estimate of effect and is likely to change the estimate.

**Very low quality:** We are very uncertain about the estimate.

^1^ Only five trials were judged to be at low risk of bias.

^2^ Moderate heterogeneity (I2 =56%) was found.

^3^ Only three trials were judged to be at low risk of bias.

^4^ High heterogeneity (I2 =86%) was found.

^5^ RR with 95% CI for one trial was 18.42 (2.49-135.95
